# Caste-based Diminished Returns of Educational Attainment on Wealth Accumulation in India

**DOI:** 10.31586/ojer.2024.1056

**Published:** 2024-09-05

**Authors:** Shervin Assari, Hossein Zare

**Affiliations:** 1 Marginalization-Related Diminished Returns (MDRs) Center, Los Angeles, CA, United States; 2 Department of Internal Medicine, Charles R. Drew University of Medicine and Science, Los Angeles, CA, United States; 3 Department of Urban Public Health, Charles R. Drew University of Medicine and Science, Los Angeles, CA, United States; 4 Department of Family Medicine, Charles R. Drew University of Medicine and Science, Los Angeles, CA, United States; 5 Department of Health Policy and Management, Johns Hopkins Bloomberg School of Public Health, Baltimore, MD, United States; 6 School of Business, University of Maryland Global Campus (UMGC), Adelphi, MD, United States

**Keywords:** Caste-based Diminished Returns, Education, Wealth, India, Minorities’ Diminished Returns, Scheduled Castes, Discrimination, Economic Disparities

## Abstract

**Background:**

Education is widely recognized as a key driver of wealth generation, providing individuals with the opportunity to enhance their socioeconomic status. However, the effectiveness of education in generating wealth varies significantly across different social groups. In the United States, research has shown that Black individuals experience weaker economic returns on education compared to their White counterparts, a phenomenon explained by the theory of Minorities’ Diminished Returns (MDRs). Although MDRs have been documented in various countries, their relevance to caste-based disparities in India remains unexplored.

**Objective:**

This study aims to investigate the caste-based diminished returns of education on wealth in India. We hypothesize that the returns on educational attainment, in terms of wealth generation, will be weaker for individuals from Scheduled Castes (SCs) compared to those from higher castes, using data from the India Demographic and Health Surveys (DHS).

**Methods:**

This study was a cross-sectional analysis of DHS -2019/2021 data from India, examining the relationship between educational attainment and wealth across different caste groups (scheduled castes and non-scheduled castes). Multivariate regression models will be employed to assess the interaction between caste and education in predicting wealth outcomes, controlling for relevant covariates such as age, gender, and region.

**Results:**

The study is expected to find that the returns on education, in terms of wealth, are significantly weaker for individuals from Scheduled Castes compared to those from higher castes. This would indicate that caste-based discrimination continues to hinder the economic progress of Scheduled Castes, even when they achieve similar levels of education as their upper-caste counterparts.

**Conclusion:**

The findings of this study will extend the MDR framework to the Indian context, demonstrating that caste-based disparities result in diminished returns on education for wealth generation. This study underscores the need for targeted policies that address the specific barriers faced by Scheduled Castes in translating educational attainment into economic success and highlights the ongoing impact of caste-based discrimination in India.

## 1. Introduction

Education is often heralded as the primary engine of wealth generation [1], providing individuals with the skills, knowledge, and credentials necessary to secure higher-paying jobs and build financial security [2]. In many societies, education serves as a key to upward social mobility, allowing individuals to improve their socioeconomic status and, consequently, their wealth [3]. However, this relationship between education and wealth is not uniformly strong across all social groups [4]. In the United States, for instance, research has consistently shown that while education is a significant predictor of wealth for most, its benefits are substantially weaker for Black individuals compared to their White counterparts [4]. This phenomenon is explained by the theory of Minorities’ Diminished Returns (MDRs) [5,6].

Minorities’ Diminished Returns (MDRs) refer to the observation that the positive effects of socioeconomic resources such as education, income, and employment are systematically weaker for marginalized and minority populations [7,8]. Initially identified in the U.S., MDRs have been shown to affect various economic and health outcomes, with Black Americans often receiving fewer benefits from their educational and economic achievements compared to White Americans [4]. The theory has since been extended and replicated in different contexts, including Mexico [9], Israel [10,11], and several European countries [12]. However, the concept of MDRs has not yet been explicitly explored in the context of India, where caste-based hierarchies introduce a unique dimension to social and economic disparities.

India’s caste system is a longstanding social hierarchy that has historically dictated access to resources, opportunities, and social mobility. This system remains deeply entrenched, influencing various aspects of life, including access to education, employment, and wealth generation. Research by scholars such as Thorat [13,14]and others has highlighted that despite similar levels of educational attainment, individuals from lower castes often do not experience the same wealth outcomes as those from higher castes. This disparity is largely attributed to persistent discrimination in the labor market, where lower-caste individuals face barriers that hinder their economic progress, even when they possess similar qualifications and capital as their upper-caste counterparts.

Ashwini Deshpande [15], a prominent Indian economist known for her extensive work on caste discrimination, inequality, and labor economics, in her book *The Grammar of Caste: Economic Discrimination in Contemporary India* [16], explores the pervasive nature of caste-based discrimination in India’s economic sphere. She examines how caste continues to influence access to resources, opportunities, and outcomes in contemporary Indian society, despite constitutional safeguards and policies aimed at promoting equality. Through a detailed analysis of various economic indicators, Deshpande highlights the persistence of economic disparities among different caste groups, particularly focusing on how Scheduled Castes face systemic disadvantages in the labor market and other areas of economic life. Her work sheds light on the structural barriers that perpetuate inequality and calls for a deeper understanding of the intersection between caste and economics in India [16].

## 2. Aim

This study aims to explore the concept of caste-based diminished returns of education on wealth in India, using data from the Demographic and Health Surveys (DHS) [17]. Specifically, it will examine whether the returns on educational attainment, in terms of wealth generation, are weaker for individuals from lower castes compared to those from higher castes. By applying the MDR framework [18,19]to the Indian context, this study seeks to contribute to the understanding of how caste-based discrimination impacts the effectiveness of education as a tool for wealth generation and to highlight the unique challenges faced by marginalized groups in India.

## 3. Methods

### Data Source:

The data for this study were obtained from the India Demographic and Health Survey (DHS) [20] conducted between 2019 and 2021. The DHS is a nationally representative survey that collects detailed information on a wide range of demographic, socioeconomic, and health-related indicators across India. For the purposes of this study, we focused on the data related to wealth, caste, and other socioeconomic variables.

### Study Population:

The study included all individuals aged 18 years and older who were surveyed in the 2019–2021 DHS [21] India dataset. Individuals with missing data on key variables were excluded from the analysis, resulting in a final sample size that is representative of the Indian adult population.

### Outcome Variable:

The primary outcome variable was poverty, operationalized as low wealth. Wealth was measured using the DHS wealth index, which is based on household asset ownership, housing characteristics, and access to basic services. Households in the lowest quintile of the wealth index were classified as being in poverty (low wealth).

### Moderator Variable:

The moderator variable was caste, which was categorized as a binary variable distinguishing between Scheduled Castes (SCs) and non-Scheduled Castes. Scheduled Castes include those historically marginalized and often referred to as “Untouchables.” Non-Scheduled Castes include all other caste groups [22].

### Control Variables:

Several covariates were included in the analysis to control for potential confounding factors. These included:

#### Age:

A continuous variable representing the age of the respondent.

#### Employment Status:

A binary variable indicating whether the respondent was employed (yes/no).

#### Religion:

A categorical variable capturing the respondent’s religious affiliation (Hindu, Muslim, Christian, Other).

#### Urbanity:

A binary variable indicating whether the respondent lived in an urban or rural area.

### Statistical Analysis:

Data analysis was conducted using Stata 18.0. The sample was described both overall and separately for Upper and Scheduled Castes. Frequencies and percentages were reported for categorical variables, while means with standard errors (SE) were reported for continuous and interval variables. Independent samples t-tests and Chi-square tests were used to compare study variables between Upper and Scheduled Castes. Pearson correlation was employed to examine the bivariate relationships of educational attainment between these groups.

Logistic regression was used to assess the association between educational attainment and low wealth (poverty), with caste as a moderating variable. An interaction term between educational attainment and caste (Scheduled Castes = 1, Upper Castes = 0) was included in the model to evaluate whether the relationship between education and wealth differed by caste. The model was adjusted for potential confounders, including age, employment status, religion, and urbanity. Results are presented as odds ratios (ORs) with 95% confidence intervals (CIs).

Interaction effects were interpreted to determine whether the returns on education, in terms of reducing the likelihood of poverty, were significantly weaker for individuals from Scheduled Castes compared to those from Upper Castes. If the odds ratio (main effect) of education was less than 1, this indicated that education reduces the odds of low wealth (poverty) overall. A positive interaction term between Scheduled Caste and education would suggest that the protective effect of education against low wealth (poverty) is smaller for Scheduled Castes compared to Upper Castes. P-values less than 0.05 were considered statistically significant.

## 3. Results

### Descriptive Data:

The study sample consisted of 273,304 individuals, with 133,347 identified as Upper Caste and 139,957 as Scheduled Caste. The distribution of educational attainment varied significantly between these groups. Among Upper Castes, 22.8% had only primary education, 54.0% had secondary education, and 23.2% had higher education. In contrast, Scheduled Caste individuals were more likely to have only primary education (40.4%) and less likely to have higher education (10.8%) (p < 0.05). The majority of Scheduled Caste individuals identified as Hindu (88.6%), compared to 72.2% among Upper Castes, with significant differences observed in religious affiliation across castes (p < 0.05). Additionally, a higher proportion of Upper Caste individuals lived in urban areas (34.3%) compared to Scheduled Castes (23.2%) (p < 0.05). The sample also showed differences in wealth, with 47.4% of Scheduled Caste individuals classified as having low wealth, compared to 22.9% of Upper Caste individuals (p < 0.05).

### Bivariate Correlations:

The bivariate correlations (Table 2) reveal significant correlations between variables across both caste groups. For Upper Caste individuals, high education was negatively correlated with low wealth (r = −.312, p < 0.001). Age was positively correlated with urban location (r = .041, p < 0.001), but negatively correlated with high education (r = −.259, p < 0.001) and low wealth (r = −.051, p < 0.001). Urban location was positively correlated with high education (r = .225, p < 0.001) and negatively correlated with low wealth (r = −.309, p < 0.001).

Among Scheduled Caste individuals, the correlations were somewhat weaker. High education was negatively correlated with low wealth (r = −.292, p < 0.001). Age was positively correlated with urban location (r = .031, p < 0.001) and negatively correlated with high education (r = −.410, p < 0.001) and low wealth (r = −.038, p < 0.001). Urban location was positively correlated with high education (r = .153, p < 0.001) and negatively correlated with low wealth (r = −.347, p < 0.001).

### Descriptive Analysis of Wealth by Education and Caste:

**Figure 1.**
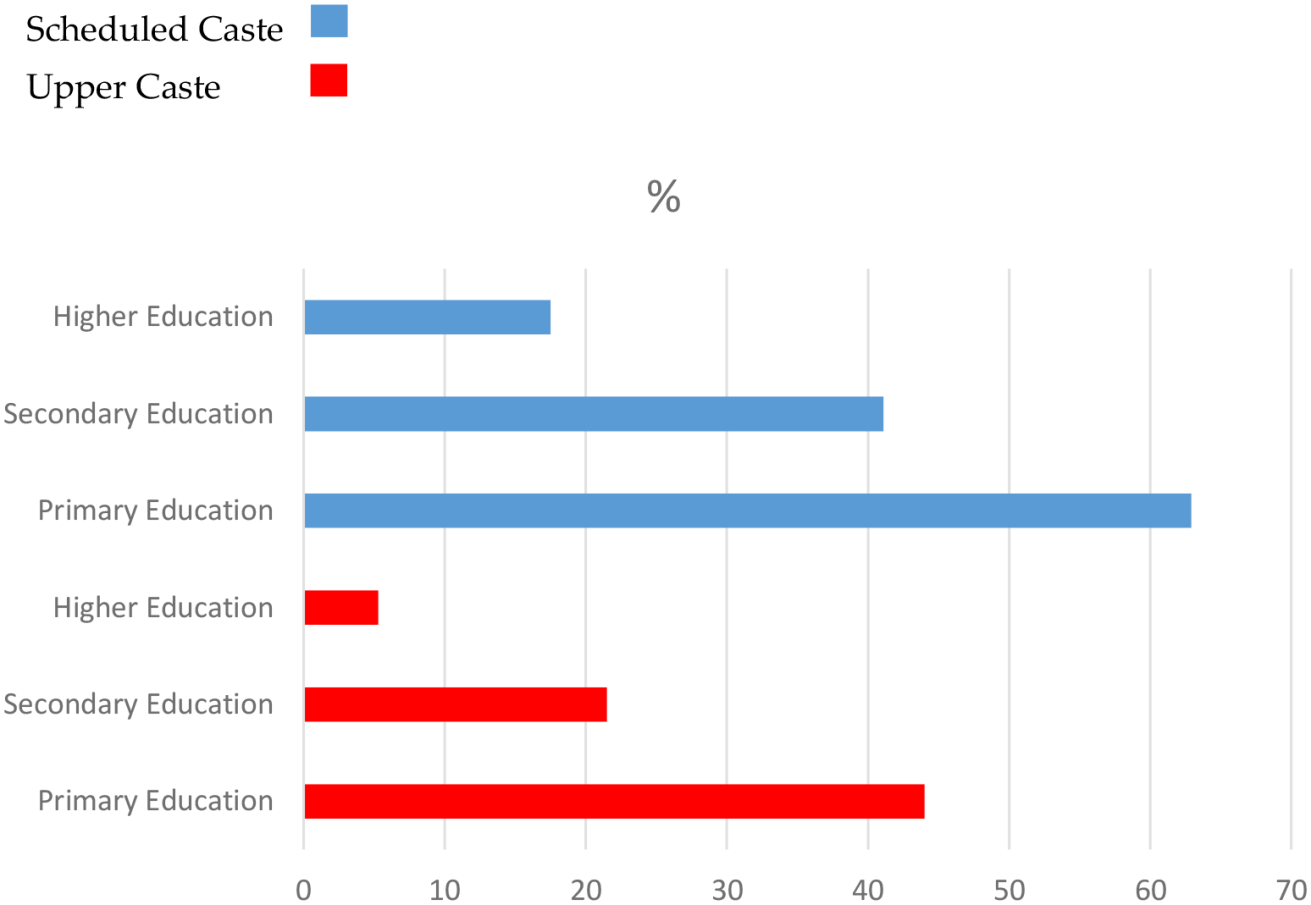
Prevalence of low wealth based on educational attainment by caste

Table 3 presents the distribution of poor wealth status by education level and caste. Among Upper Caste individuals, the percentage of those in poor wealth decreases significantly with higher education: 44.0% of those with primary education, 21.5% of those with secondary education, and 5.3% of those with higher education are classified as having poor wealth. In contrast, the pattern for Scheduled Caste individuals is less favorable, with 62.9% of those with primary education, 41.1% of those with secondary education, and 17.5% of those with higher education classified as having poor wealth. This suggests that while education is associated with improved wealth outcomes, the returns on education are significantly lower for Scheduled Castes compared to Upper Castes, further indicating the persistence of caste-based disparities in economic outcomes (Figure 1).

### Multivariable Models

The results from four logistic regression models are presented in Tables 4 and 5. These models examine the association between caste, education, and location (urban vs. rural) on the outcome variable, with a particular focus on the interaction between caste and education.

### Main Effect Model (Model 1):

In the main effect model (Model 1), age, caste, education level, and urban location were significant predictors of the outcome variable. The odds of the outcome decreased with increasing age (OR = 0.958, 95% CI = 0.957–0.959, p < .001). Belonging to a Scheduled Caste was associated with significantly higher odds of the outcome compared to Upper Caste individuals (OR = 2.257, 95% CI = 2.215–2.299, p < .001). Higher education levels were protective, with individuals having secondary education showing an OR of 0.264 (95% CI = 0.258–0.270, p < .001) and those with higher education showing an OR of 0.078 (95% CI = 0.075–0.081, p < .001). Living in an urban location was also significantly associated with lower odds of the outcome (OR = 0.148, 95% CI = 0.144–0.152, p < .001).

### Interaction Model (Model 2):

When the interaction between caste and education was introduced in Model 2, the association between Scheduled Caste and the outcome remained strong, though slightly attenuated (OR = 2.111, 95% CI = 2.048–2.176, p < .001). The protective effect of education was still evident, but the interaction terms revealed that the protective effect of education was less pronounced for Scheduled Caste individuals. Specifically, the interaction between secondary education and Scheduled Caste was associated with a slight increase in the odds of the outcome (OR = 1.071, 95% CI = 1.030–1.114, p < .001), and the interaction between higher education and Scheduled Caste was associated with an even greater increase in the odds (OR = 1.449, 95% CI = 1.346–1.559, p < .001). These findings suggest that while higher education is generally protective, its protective effects are diminished for individuals belonging to Scheduled Castes.

### Caste-Specific Models (Model 3 and Model 4)

The caste-specific models further elucidate these relationships. In the model restricted to Upper Caste individuals (Model 3), age, education level, and urban location were all significant predictors of the outcome. The protective effects of secondary and higher education were strong (OR = 0.255, 95% CI = 0.246–0.264, p < .001 for secondary education; OR = 0.064, 95% CI = 0.060–0.068, p < .001 for higher education). Additionally, living in an urban area was associated with significantly lower odds of the outcome (OR = 0.134, 95% CI = 0.128–0.140, p < .001).

In the model restricted to Scheduled Caste individuals (Model 4), the pattern was similar but with some notable differences. Age, education, and urban location remained significant predictors. However, the protective effect of higher education was less pronounced for Scheduled Caste individuals compared to Upper Caste individuals (OR = 0.091, 95% CI = 0.087–0.096, p < .001). This suggests that while higher education is associated with reduced odds of the outcome in both caste groups, the reduction is less significant among Scheduled Castes.

## 4. Discussion

The primary aim of this study was to investigate the concept of caste-based diminished returns of education on wealth in India, using data from the 2019–2021 Demographic and Health Survey (DHS). Specifically, the study sought to determine whether the association between educational attainment and the likelihood of escaping poverty was moderated by caste, with a focus on Scheduled Castes (SCs) versus non-Scheduled Castes. The study controlled for potential confounders, including age, employment status, religion, and urbanity.

The results of our analysis indicate that the returns on education, in terms of reducing the odds of poverty, are significantly weaker for individuals from Scheduled Castes compared to those from non-Scheduled Castes. While higher education generally reduces the odds of being in poverty, this protective effect is less pronounced among Scheduled Castes, even after controlling for age, employment status, religion, and urbanity. This finding aligns with the theory of Minorities’ Diminished Returns (MDRs)[23,24], suggesting that systemic and structural barriers continue to limit the economic benefits that Scheduled Castes can derive from educational attainment.

In her book *The Grammar of Caste: Economic Discrimination in Contemporary India*, Ashwini Deshpande [16] describes and discusses the persistent and systemic nature of caste-based discrimination in India’s economic structures. She examines how caste continues to shape access to opportunities in labor markets, education, and business, arguing that these discriminatory practices are not just historical remnants but ongoing realities that contribute to the entrenched poverty and inequality among lower castes, especially Dalits. Deshpande emphasizes the critical need for more effective policies and interventions to address these deep-rooted economic disparities and achieve social justice in India [25].

The persistence of graded caste inequality in India remains a deeply entrenched issue, despite various efforts to address it. Other scholars have written extensively on the topic of caste-based discrimination in India. As highlighted by Thorat and Madheswaran, the caste system is not simply a matter of being rich or poor; it is a more complex and rigid structure where privileges decrease as one moves down the caste hierarchy, with Brahmins at the top enjoying the most rights, and Dalits, or “untouchables,” at the bottom, suffering the greatest deprivation. This inequality is a fundamental feature of the caste system, which hierarchically organizes society based on the unequal distribution of rights and privileges among different caste groups [13].

Most research on inequality in income and human development indicators has focused on differential access to education, employment, and occupations across different caste groups. This research has shown that wealth distribution in India is highly skewed, with higher castes owning a disproportionate share of the country’s wealth compared to lower castes, particularly Dalits, who own far less than their population share would suggest. This unequal ownership of wealth directly translates into unequal opportunities in employment and education, further perpetuating the cycle of poverty and discrimination [13]. However, such disparities continue even after addressing disparities in asset ownership and access to education.

Disparities within the same educational categories, however, are not mainly due to access to resources but the systematic marginalization of lower castes across all educational levels. Caste discrimination in the labor market plays a significant role in maintaining this inequality across castes with similar education. Systemic discrimination against Dalits in wages, job opportunities, and occupational segregation are all well-documented, with evidence showing that Dalits are often paid less than their higher-caste counterparts for the same work and are disproportionately represented in low-paying, casual labor. This discrimination, which limits the upward mobility of Dalits and other lower castes, serves to maintain the economic and social privileges of the higher castes [13].

The persistence of caste-based discrimination, despite legal protections and affirmative action policies, raises important questions about the effectiveness of these measures and the deep-rooted nature of caste prejudice in Indian society. Theories of discrimination, including those proposed by Becker and Arrow, suggest that discrimination is driven by both individual and group interests, where dominant groups seek to maintain their status and material advantages through discriminatory practices. In the context of India, this is further compounded by the ideological support provided by religious and cultural norms, which sanctify the caste system and the associated graded inequalities [13].

Thorat and Madheswaran analyzed Indian economic data and showed that the ancient system of graded caste inequality continues to persist in modern India, particularly in terms of economic well-being. The Monthly Per Capita Consumption Expenditure (MPCE) highlights this disparity, with higher castes (HCs) enjoying an average MPCE of ₹2,413, compared to ₹1,294 for Scheduled Castes (SCs). This graded inequality is further reflected in poverty rates, where only 9% of HCs are poor, as opposed to 20% of OBCs and 30% of SCs, who remain at the bottom of the caste hierarchy. The high incidence of poverty among SCs can be attributed to their limited ownership of wealth and human capital. In 2013, HCs owned nearly 45% of the country’s wealth—more than twice their population share—while Scheduled Castes owned only 7%, which is significantly less than their 18% population share. These disparities in wealth ownership directly contribute to the continued economic marginalization of Scheduled Castes, underscoring the persistent nature of caste-based inequality in India [13].

The causes of this graded inequality extend beyond wealth ownership to include disparities in education and employment opportunities. In 2012, 52% of people were self-employed as farmers and entrepreneurs, but SCs were more dependent on wage labor, with 44% engaged in this form of work compared to 26% of OBCs and just 11% of HCs. Discrimination in the labor market further exacerbates these inequalities, particularly for SCs, who face significant barriers in accessing higher-paying jobs and securing fair wages. For instance, in the regular labor market, 71.5% of the wage gap between SCs and HCs can be attributed to differences in endowment factors like education and capital assets, but 28.5% of the gap is due to caste discrimination. This discrimination is especially pronounced in the private sector, where the wage gap attributable to caste discrimination is higher than in the public sector, and it increases at higher levels of wage distribution. Thus, the combination of historical and ongoing discrimination in wealth, education, and employment continues to perpetuate caste-based inequalities in India, making it a deeply entrenched social issue [13].

However, the issue of graded caste inequality in India is complex and multifaceted, deeply embedded in both the economic structures and the cultural fabric of the society [13]. Addressing this inequality requires not only economic reforms but also a fundamental shift in the social and cultural attitudes that sustain caste-based discrimination. Without such changes, the entrenched disparities in wealth, education, and employment between different caste groups are likely to persist, continuing to disadvantage the most marginalized communities in Indian society [13].

### Implications:

The findings of this study have important implications for policy and practice. They highlight the persistence of caste-based economic disparities in India, even in the context of educational attainment. This suggests that policies solely focused on increasing access to education may be insufficient to address poverty among marginalized groups. There is a need for targeted interventions that address the structural barriers and discriminatory practices in the labor market that disproportionately affect Scheduled Castes. Additionally, these findings underscore the importance of considering caste as a critical factor in the design and evaluation of poverty alleviation programs.

### Limitations:

Despite the strengths of this study, there are several limitations that should be acknowledged. First, the cross-sectional nature of the DHS data limits the ability to make causal inferences about the relationship between education, caste, and poverty. Second, the study relies on self-reported data, which may be subject to reporting biases. Third, the DHS wealth index, while widely used, is a proxy measure of poverty and may not capture the full complexity of wealth and economic status. Finally, the study did not explore other potential moderators or mediators, such as regional differences or variations in social capital, which could also influence the relationship between education and wealth.

### Future Research:

Future research should explore longitudinal data to better understand the causal pathways between education, caste, and wealth over time. Additionally, qualitative studies could provide deeper insights into the lived experiences of Scheduled Castes and the specific challenges they face in translating educational attainment into economic success. Research could also examine the role of other factors, such as social networks, regional policies, and access to credit, in shaping the returns on education for different caste groups. Expanding the analysis to include other marginalized groups, such as Scheduled Tribes and Other Backward Classes, could further enrich the understanding of caste-based economic disparities.

## 5. Conclusion

This study contributes to the growing body of literature on Minorities’ Diminished Returns (MDRs) by extending the concept to the Indian context. The findings demonstrate that individuals from Scheduled Castes in India experience weaker economic returns on education compared to their non-Scheduled Caste counterparts, underscoring the ongoing impact of caste-based discrimination within both the education system and the labor market. These results underscore the need for comprehensive policies that address structural barriers that limit both educational access and economic mobility for marginalized groups in India. By prioritizing equity in education and the labor market, policymakers can work towards reducing poverty and advancing social justice in India.

## Figures and Tables

**Table 1.** Descriptive Data Overall and by Caste (n = 273,304)

	All		Upper Caste (n = 133347)		Scheduled Caste (n = 139957)	
	n	%	n	%	n	%
Education*						
Primary	86901	31.8	30391	22.8	56510	40.4
Secondary	140370	51.4	72021	54.0	68349	48.8
Higher	46033	16.8	30935	23.2	15098	10.8
Scheduled Caste						
No	133347	48.8	133347	100.0	-	-
Yes	139957	51.2	-	-	139957	100.0
Religion*						
Hindu	220377	80.6	96307	72.2	124070	88.6
Muslim	29971	11.0	26930	20.2	3041	2.2
Christian	5300	1.9	2572	1.9	2728	1.9
Sikh	13180	4.8	5957	4.5	7223	5.2
Buddhist / Neo-Buddhist	2960	1.1	382	.3	2578	1.8
Jain	608	.2	578	.4	30	.0
Jewish	2	.0	2	.0	0	.0
Parsi / Zoroastrian	30	.0	3	.0	27	.0
No religion	34	.0	14	.0	20	.0
Other	842	.3	602	.5	240	.2
Urban*						
No	195131	71.40	87631	65.7	107500	76.8
Yes	78173	28.60	45716	34.3	32457	23.2
Location / State*						
Jammu & Kashmir	13971	5.1	12029	9.0	1942	1.4
Himachal Pradesh	7562	2.8	4963	3.7	2599	1.9
Punjab	17965	6.6	7859	5.9	10106	7.2
Chandigarh	604	.2	401	.3	203	.1
Uttarakhand	9954	3.6	7146	5.4	2808	2.0
Haryana	14941	5.5	8419	6.3	6522	4.7
Nct Of Delhi	8094	3.0	5271	4.0	2823	2.0
Rajasthan	16241	5.9	7123	5.3	9118	6.5
Uttar Pradesh	40819	14.9	17918	13.4	22901	16.4
Bihar	16878	6.2	6560	4.9	10318	7.4
Sikkim	333	.1	145	.1	188	.1
Arunachal Pradesh	2396	.9	1056	.8	1340	1.0
Nagaland	209	.1	19	.0	190	.1
Manipur	2739	1.0	2212	1.7	527	.4
Mizoram	226	.1	1	.0	225	.2
Tripura	2378	.9	750	.6	1628	1.2
Meghalaya	439	.2	243	.2	196	.1
Assam	8312	3.0	3963	3.0	4349	3.1
West Bengal	12072	4.4	5820	4.4	6252	4.5
Jharkhand	6282	2.3	2167	1.6	4115	2.9
Odisha	10016	3.7	4294	3.2	5722	4.1
Chhattisgarh	4422	1.6	1050	.8	3372	2.4
Madhya Pradesh	14276	5.2	6353	4.8	7923	5.7
Gujarat	9338	3.4	5215	3.9	4123	2.9
Dadra & Nagar Haveli And Daman & Diu	718	.3	431	.3	287	.2
Maharashtra	16058	5.9	10275	7.7	5783	4.1
Andhra Pradesh	4640	1.7	2225	1.7	2415	1.7
Karnataka	8463	3.1	2047	1.5	6416	4.6
Goa	663	.2	534	.4	129	.1
Lakshadweep	78	.0	47	.0	31	.0
Kerala	4072	1.5	2791	2.1	1281	.9
Tamil Nadu	7626	2.8	393	.3	7233	5.2
Puducherry	928	.3	282	.2	646	.5
Andaman & Nicobar Islands	1145	.4	1129	.8	16	.0
Telangana	8359	3.1	2167	1.6	6192	4.4
Ladakh	87	.0	49	.0	38	.0
Low Wealth*						
No	176557	64.6	102873	77.1	73684	52.6
Yes	96747	35.4	30474	22.9	66273	47.4

* p<0.05 for comparison of Castes

**Table 2.** Bivariate Correlations of Education and Wealth by Caste

			1	2	3	4
	**Upper Caste** (**n** = 133,347)					
	1 Age	r	1			
		p				
				
	2 Location (Urban)	r	.041**	1		
		p	<0.001			
				
	3 (High Education 1–3)	r	−.259**	.225**	1	
		p	<0.001	<0.001		
				
	4 Wealth (Low)	r	−.051**	−.309**	−.312**	1
		p	<0.001	0.000	0.000	
				
	**Schedule Caste** (**n** = 139,957)					
	1 Age	r	1			
		p				
				
	2 Location (Urban)	r	.031**	1		
		p	<0.001			
				
	3 (High Education 1–3)	r	−.410**	.153**	1	
		p	<0.001	<0.001		
				
	4 Wealth (Low)	r	−.038**	−.347**	−.292**	1
		p	<0.001	<0.001	<0.001	
				

Pearson Correlation Test;

** p < 0.001

**Table 3.** Descriptives of Poor Wealth by Intersection of Education and Caste

Caste	Education	n	%
			
Upper Caste	Primary Education	13,364	44.0
Upper Caste	Secondary Education	15,470	21.5
Upper Caste	Higher Education	1,640	5.3
			
Scheduled Caste	Primary Education	35,555	62.9
Scheduled Caste	Secondary Education	28,071	41.1
Scheduled Caste	Higher Education	2,647	17.5

**Table 4.** Logistic Regression Model Without and With Intersection of Education and Caste

	Beta	SE	OR	95% CI		Sig
**Main Effect Model (Model 1)**						
Age	−.043	.001	.958	.957	.959	< .001
Scheduled Caste	.814	.009	2.257	2.215	2.299	< .001
Education						< .001
Primary						
Secondary	−1.332	.011	.264	.258	.270	< .001
Higher	−2.554	.019	.078	.075	.081	< .001
Location (Urban)	−1.913	.014	.148	.144	.152	< .001
Constant	1.631	.022	5.107			< .001
**Interaction Model (Model 2)**						
Age	−.043	.001	.958	.957	.959	< .001
Scheduled Caste	.747	.016	2.111	2.048	2.176	< .001
Education						< .001
Primary						
Secondary	−1.374	.017	.253	.245	.261	< .001
Higher	−2.762	.029	.063	.060	.067	< .001
Education × Caste						< .001
Secondary Education × Scheduled Caste	.069	.020	1.071	1.030	1.114	< .001
Higher Education × Scheduled Caste	.371	.038	1.449	1.346	1.559	< .001
Location Urban	−1.910	.014	.148	.144	.152	< .001
Constant	1.669	.024	5.309			< .001

**Table 5.** Logistic Regression Model Within Each Caste

	Beta	SE	OR	95% CI		Sig
**Upper Caste (Model 3)**						
Age	−0.042	0.001	0.959	0.957	0.960	< .001
Education						< .001
Primary						
Secondary	−1.368	0.018	0.255	0.246	0.264	< .001
Higher	−2.751	0.030	0.064	0.060	0.068	< .001
Location (Urban)	−2.013	0.023	0.134	0.128	0.140	< .001
Constant	1.651	0.033	5.213			< .001
						
**Scheduled Caste (Model 4)**						
Age	−0.044	0.001	0.957	0.956	0.959	< .001
Education						< .001
Primary						
Secondary	−1.311	0.015	0.270	0.262	0.278	< .001
Higher	−2.397	0.026	0.091	0.087	0.096	< .001
Location (Urban)	−1.852	0.017	0.157	0.152	0.162	< .001
Constant	2.431	0.028	11.375			< .001
